# Supplementary material for: PiE: an open-source pipeline for home cage behavioral analysis
Source: Front Neurosci. 2023 Jul 31;17:1222644. doi: 10.3389/fnins.2023.1222644 (PMC10423934; doi:10.3389/fnins.2023.1222644)
Supplement: Supplementary file 1 [file Data_Sheet_1.PDF]

Materials and Equipment

Product	Price at Publication	Notes	Link
Slotted Rails for Box Frames	Project dependent		https://8020.net/20-2020.html
ABS Panels for Box Walls	Project dependent		https://8020.net/65-2613-s.html
Transparent mouse cage bottom	Project dependent		https://www.altdesign.com/mouse-cages/
Acrylic for mouse cage tops	Project dependent	Optically transparent	https://8020.net/2602.html
Raspberry Pi 4 - 4GB RAM	55.00	This has a list of places to buy the latest Raspberry Pi	https://www.raspberrypi.org/products/raspberry-pi-4-model-b/?variant=raspberry-pi-4-model-b-4gb
MicroSD for Raspberry Pi OS	9.95		https://a.co/3xWn2iS
USB for video storage	14.69		https://a.co/d/a0OOtEt
Raspberry Pi NoIR Camera Board	29.95	V2 - 8 Megapixels available at time of publication	https://www.adafruit.com/product/3100
Camera flex cable	3.95	This cable is preferable to the white one that comes with the camera because you want to minimize reflective surfaces inside the box	https://www.adafruit.com/product/2143
4-channel relay	8.99		https://a.co/d/9MSxh6g
Jumper cables M-M	1.95		https://www.adafruit.com/product/1956?gclid=Cj0KCQiAyoeCBhCTARIsAOfpKxgBxDelmI_MgZMxnHIIXkebpgL05hoSPHO7N2HIXqMe3hODA3IntfoaArIuEALw_wcB
Jumper Wires F-F	3.95		https://www.adafruit.com/product/266
Jumper Wires F-M	3.95		https://www.adafruit.com/product/826
Hook-up wire	22.50	Stranded wire is more flexible - solid core wire can be used here but we found stranded wire to be easier to solder.	https://www.sparkfun.com/products/11375
Wire stripping tool	7.99		https://a.co/d/13VGGXO
Soldering iron	54.75		https://a.co/d/9kCbROy
Temperature Humidity Sensor	7.95		https://www.adafruit.com/product/5181
Power cords	8.99	For use from power source to Raspberry Pi and from power source to Quad Relay	https://a.co/d/ce8aGa2
Wall power	25.99	For Raspberry Pis and Quad Relays	https://a.co/d/6WzYNTQ
Mirrors	12.00		https://a.co/d/7w8RPo8
Mirror adjustment stand	4.95	Test your configuration dimensions before building the exterior of the behavior box	https://www.adafruit.com/product/1679
Fan	12.66		https://www.digikey.com/en/products/detail/qualtek/FAD1-06025BBHW12-A/7724541
Mounting Hardware	14.95		https://www.adafruit.com/product/3658
Ethernet Cables	18.49		https://a.co/d/6SkX2uE
Router	59.99		https://a.co/d/6ggbFR1
Multi-port switch box	43.99		https://a.co/d/9DOntxV
**Lighting:**	There are three different lighting options, outlined below:		
**Option 1:**	Rigid LED bars	More expensive, Least Effort, Least Flexible	
White lights	29.99		https://a.co/d/bJXB9DL
Infrared Lights	15.95	Note: These are 20" so you would need a box at least 21" wide for these to fit.	https://ledlightsworld.com/collections/ir-infrared-led-strips/products/12vdc-waterproof-ip65-smd3528-36-ir-infrared-850nm-940nm-led-linear-rigid-strip-36leds-3-6w-per-piece
**Option 2:**	LED Strips	Middle Expense, Middle Effort, Middle Flexibility	
White light strips	13.99		https://a.co/d/8w5TzOP
Infrared LED strips	35	This single reel would cover multiple boxes so it's more cost effective for multi-box projects than Option 1 but may be less cost-effective if the user is building a single box.	https://ledlightsworld.com/collections/ir-infrared-led-strips/products/dc12v-smd3528-300-ir-infrared-850nm-940nm-single-chip-flexible-led-strips-60leds-4-8w-per-meter?variant=17836968476762
**Option 3:**	Individual LEDs	Lowest expense, most effort, most flexibility	Individually soldered
Printed circuit board	2.99		https://www.superbrightleds.com/moreinfo/resistors/universal-9-led-pcb-sbl-pcb1/548/1743/
White light LEDs	6.95		https://www.adafruit.com/product/754?gclid=Cj0KCQiAyoeCBhCTARIsAOfpKxjF0gxa_VYT2nrAHeXsAAEzSJVIRxnQMbOP_eWktV09xGSgnAH3jzcaAhiWEALw_wcB
Infrared LEDs	10.89		https://a.co/d/3pAPUmw
Splicers	19.96	User needs 4 per box, this is the price for 50	https://a.co/d/7MjUhzW
Resistors	0.95		https://www.sparkfun.com/products/14490
Optional Upgrades:			
Mirror adjustment platform	71.96	Optional	https://www.thorlabs.com/newgrouppage9.cfm?objectgroup_id=10660
Soldering workbench	39.99	Optional	https://a.co/d/b9er7TP
Camera tripod mount case	2.95	Optional	https://www.adafruit.com/product/3253
Tripod mount	9.84	Optional	https://a.co/d/ihrs3sG
Forced air vents:	If active air exchange is preferred, an aquarium pump can be used to route airflow into the boxes through rubber grommets in the panels, with another tube and grommet on the opposite side of the box as an outflow vent		
Aquarium pump	14.99	Optional	https://a.co/d/5mFpjFG
Rubber grommets	6.99	Optional	https://a.co/d/dOoeeqe
Black tubing	23.78	Optional	https://a.co/d/8YI0CPT
